# Case Report: Response to ivosidenib in patients with cholangiocarcinoma: a clinical perspective with illustrative cases

**DOI:** 10.3389/fonc.2026.1617664

**Published:** 2026-06-02

**Authors:** Yannick Buccella

**Affiliations:** Medical Oncology and Haematology Unit, Stadtspital Triemli, Zürich, Switzerland

**Keywords:** cholangiocarcinoma, *IDH1* mutations, ivosidenib, targeted therapy, tumor response

## Abstract

Cholangiocarcinoma (CCA) is a rare cancer, rich in molecular targets. Molecular targeted therapy is the recommended second-line approach for patients with advanced or metastatic CCA, including ivosidenib for patients with mutations in the gene for isocitrate dehydrogenase 1 (*IDH1).* Herein, three case studies of patients with CCA who received ivosidenib are presented, to illustrate key issues relevant to the use of this treatment in clinical practice. The first case demonstrates a durable response to ivosidenib treatment lasting >1 year, as seen in the phase 3 ClarIDHy study, where most responders had stable disease and the presence of a response significantly prolonged survival, particularly in those with a response lasting >1 year (2-year survival rate of 92%). The second case illustrates tumor shrinkage with ivosidenib, which was seen in only 2% of patients in the ClarIDHy study. This patient had both *IDH1* and human epidermal growth factor receptor 2 (*HER2*) mutations, providing more than one targeted treatment option, but chose oral therapy with ivosidenib. The third case describes some of the challenges when managing CCA in older patients, particularly if performance status (PS) is impaired. Because of the patient’s advanced age and frailty, she began first-line ivosidenib as off-label treatment. Although she experienced significant improvement in PS, she developed malignant hypercalcemia. The risk of hypercalcemia may be higher in older patients and those with *IDH1* mutations. These cases illustrate the potential benefits of ivosidenib, in terms of clinical response, quality of life, and tolerability. They also highlight the importance of obtaining adequate biopsy samples for molecular testing at diagnosis to identify potential targetable mutations. Performing molecular testing (in the absence of adequate tumor tissue with liquid biopsy) as early as possible is already an integral part of the treatment pathway planning for CCA patients. Early molecular testing may therefore lead to the possibility of administering targeted therapy in the first-line setting within this patient group, pending the outcomes of clinical trials.

## 1 Introduction

The term cholangiocarcinoma (CCA) is used to describe a diverse group of rare malignancies that arise in the biliary tract and are broadly subdivided into intrahepatic CCA (iCCA; develop in the bile ducts or segmental ductules) or extrahepatic (eCCA; develop in the common bile duct or close to its junction) (1). The incidence of CCA is high in some parts of East Asia, where there are endemic liver fluke infestations. In most parts of the world, the incidence is increasing in line with the growing prevalence of risk factors for liver disease, including viral hepatitis, diabetes, and alcohol use (1).

If identified early, CCA can be surgically resected with curative intent (2). However, the majority of patients with CCA are diagnosed at an advanced stage [because early CCA is usually asymptomatic or associated with nebulous symptoms (1)] and will require systemic therapy. The European Society for Medical Oncology (ESMO) guidelines recommend cisplatin + gemcitabine (± durvalumab), as first-line therapy for locally advanced and metastatic CCA (2). For patients who progress after first-line chemotherapy, the recommended approach is either molecular targeted therapy (based on the patient’s prevailing genetic mutation) or second-line chemotherapy (for those without a relevant CCA driver mutation) (2, 3).

CCA is a tumor rich in molecular targets (4–9). Common targetable genetic alterations in both iCCA and eCCA include Kirsten rat sarcoma viral oncogene homolog (*KRAS*), tumor protein 53 (*TP53*), and Mothers against decapentaplegic homolog 4 (*SMAD4*) (1). Targets that are much more frequent in iCCA than eCCA include fibroblast growth factor receptor 2 (*FGFR2*) fusions and mutations in the genes for isocitrate dehydrogenase 1 and 2 (*IDH1/2*), v-Raf murine sarcoma viral oncogene homolog B (*BRAF*), breast cancer gene 1-associated protein 1 (*BAP1*), and *ARID1A* (that encodes adenine- and thymine-rich interacting domain, containing protein 1A) (1, 4, 6). Targetable mutations that are common in eCCA but not iCCA are human epidermal growth factor receptor 2 (*HER2*), Claudin 18.2, fusions in the *PRKACA* and *PRKACB* genes encoding for the Cα and Cβ subunits on cyclic AMP-dependent protein kinase, and mutations in human epidermal growth factor receptor 3 (*ERBB3*) and E74-like ETS transcription factor 3 (*ELF3*) (1, 5). Other less common mutations in CCA include those of rearranged during transfection (*RET*), neuregulin 1 (*NRG1*), neurotrophic tyrosine receptor kinase (*NTRK*), mouse double minute 2 (*MDM2*), and Breast CAncer gene 1/2 (*BRCA1/2*) genes (7, 9). Molecular targeted therapies are available for many of these driver mutations. The relevant driver mutations in CCA, their associated molecular targeted treatments, and the evidence level defined in ESMO guidelines are summarized in **Supplementary Table S1** (3).

Mutations in the *IDH1* gene are some of the most common molecular alterations in iCCA (10). Treatment with the IDH1 inhibitor ivosidenib is recommended in patients with *IDH1* mutations (2, 3), based on data from the phase 3 ClarIDHy study, which demonstrated an increased duration of progression-free survival (PFS; primary endpoint) in patients with advanced *IDH1*-mutated CCA receiving once-daily ivosidenib (11). Significant improvements were also seen in secondary endpoints, including overall survival (OS) after adjustment for crossover from placebo to ivosidenib treatment (11, 12). Some patients in the ClarIDHy study received treatment for a prolonged duration (maximum of 34.4 months) (12), indicating that a number of patients had a durable response to ivosidenib.

This article describes three patients with CCA who received ivosidenib in real-world clinical practice to illustrate factors that are relevant to the practical use of ivosidenib in patients with *IDH1*-mutated CCA. Comment is provided on the cases, in the context of relevant literature and the clinical expertise of the author. The timelines for treatment in each case is summarized in **Figure 1**.

**Figure 1:**
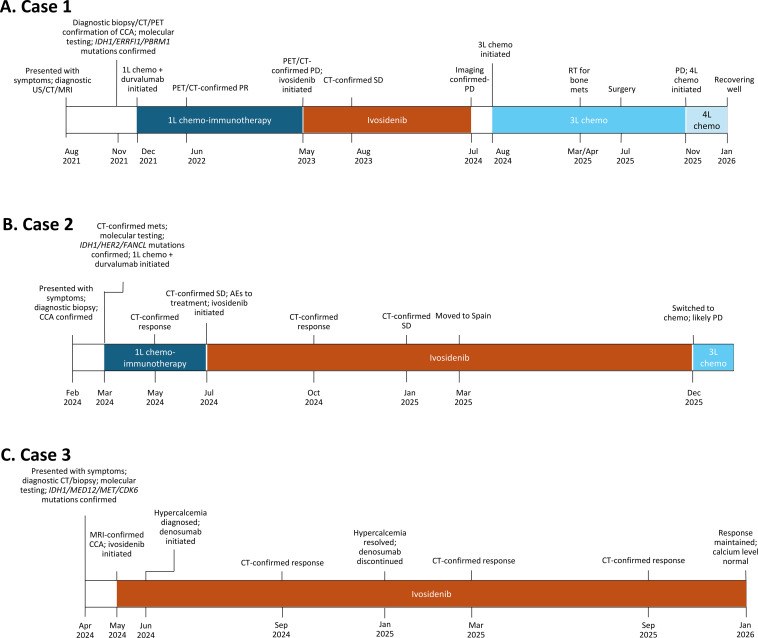
Timelines for **(A)** case 1, **(B)** case 2 and **(C)** case 3. AEs, adverse events; CCA, cholangiocarcinoma; *CDK6*, cyclin dependent kinase 6 gene; chemo, chemotherapy; CT, computed tomography; PD, progressive disease; *ERRFI1*, erythroblastic leukemia viral oncogene receptor feedback inhibitor 1 gene; *FANCL*, Fanconi anemia complementation group L gene; *HER2*, human epidermal growth factor receptor 2 gene; *IDH1*, isocitrate dehydrogenase 1 gene; L, line; *MED12*, mediator complex subunit 12 gene; mets, metastases; MRI, magnetic resonance imaging; *PBRM1*, polybromo-1 gene; PET, positron emission tomography; PR, partial response; RT, radiotherapy; SD, stable disease; US, ultrasound.

All the patients provided written informed consent for their clinical information to be presented in this article.

## 2 Case 1

A 66-year-old Caucasian woman presented in August 2021 with a 2-year history of upper abdominal discomfort, accompanied in the prior several months by mild hepatitis B virus-like symptoms (e.g., night sweats, slight weight loss, significant fatigue). She also had osteoporosis and experienced migraines but had no other relevant medical history. Abdominal ultrasound showed a large mass in the right hepatic lobe and another lesion in the hepatic hilum or at the head of the pancreas. Computed tomography (CT) and magnetic resonance imaging (MRI) showed multiple, partially confluent liver lesions, but no discernible primary tumor. There was extensive lymph node involvement in the hepatic hilum, adjacent to supra-aortic nodes (along the celiac trunk), in the retroperitoneum and between the aorta and inferior vena cava.

In November 2021, a laparoscopic liver biopsy showed adenocarcinoma cells with a pancreatobiliary subtype. Further CT and positron emission tomography (PET) imaging confirmed the presence of lobulated lesions across hepatic segments V and VI–IVb, with peripheral intense metabolic activity and multiple highly metabolically active satellite lesions with central necrosis. There were also multiple lymph nodes suspicious for metastases in the liver hilum, along the celiac trunk and in the retroperitoneal, para-aortic, mediastinal (level 2L), and left hilar regions. Molecular testing showed an R132 mutation in *IDH1*, an S163fs*13 mutation in the erythroblastic leukemia viral oncogene receptor feedback inhibitor 1 (*ERRFI1*) gene and an E967* mutation in the polybromo-1 (*PBRM1*) gene.

Her tumor was inoperable; therefore, systemic chemotherapy with cisplatin + gemcitabine was started in December 2021. Durvalumab was added after the publication of the TOPAZ-1 study results, which showed the benefit of adding immunotherapy to first-line chemotherapy in CCA (13). After completion of 6 months’ chemotherapy, in June 2022, PET/CT imaging showed a partial response (PR), with subtotal morphological regression and total metabolic regression of the peritoneal metastases; stability in the size of lymph node metastases in the hilum, celiac region, and retroperitoneum, as well as a >30% reduction in metabolic activity; size-stable and metabolically unchanged lymph nodes in the upper mediastinum and left hilar regions; and no relevant change in the size or metabolic activity of the iCCA tumors.

Maintenance durvalumab therapy was continued until May 2023, when PET/CT imaging showed hepatic and lymphatic progression. Ivosidenib was started and CT imaging 3 months later showed stable disease (SD), with no significant change in the diameter of the target liver lesion. Ivosidenib was well tolerated, and the patient was able to keep working and travelling for >1 year on ivosidenib until July 2024, when imaging showed hepatic and lymphatic progression and a new solitary metastasis in a peritoneal lymph node. In August 2024, she began third-line chemotherapy (a rechallenge with cisplatin-based chemotherapy). The patient did not receive further treatment until March/April 2025 when she received radiotherapy for focal symptomatic bone metastases and July 2025 when she underwent surgery. In November 2025, systemic disease progression occurred (new bone, lymphatic, and peritoneal metastases). Currently, the patient is undergoing fourth-line treatment with FOLFOX and has recovered well from previous treatment, having an Eastern Cooperative Oncology Group performance status (ECOG PS) score of 1.

### 2.1 Comment

This case is illustrative of the type of response seen in participants of the ClarIDHy study, where the objective response rate (ORR) was 2% (with 3/124 patients achieving PR), but the disease control rate (DCR) was 53% because another 63 patients had SD (11). The pattern of response to ivosidenib in the ClarIDHy study was notable in a number of ways. First, the Kaplan–Meier curves for PFS with ivosidenib and placebo did not show any marked separation until after about 2 months of treatment, at which time about 40% of patients in both groups had met the primary endpoint (11). As a result, there was a relatively short median PFS in the ivosidenib group (2.7 months) that was heavily influenced by those patients who progressed quickly. On the other hand, many of the patients who did not progress within the first 2 months had a prolonged course of SD, whereas all of the participants in the placebo arm had disease progression at 6 months. Therefore, the clear benefit of ivosidenib versus placebo is better illustrated by the hazard ratio (HR) for PFS (0.37 [95% confidence interval (CI) 0.25–0.54]) and 6-month PFS rate (32% vs 0%) than it is by a comparison of median PFS between the groups (2.7 vs 1.4 months) (11).

The final OS data from ClarIDHy were affected by patients who crossed over from the placebo to the ivosidenib arm; therefore, the data were statistically adjusted using the rank-preserving structural failure time (RPFST) technique (14) to account for this. After RPFST adjustment, median OS was 10.3 versus 5.1 months with ivosidenib versus placebo (HR 0.49 [95% CI 0.34–0.70]; p<0.001) (12).

Because some patients have a durable response to ivosidenib, a *post hoc* analysis was undertaken to compare outcomes in the 26 patients who received ivosidenib for ≥1 year (19 randomized to ivosidenib from the start and seven who had crossed over to ivosidenib from the placebo arm) versus the 140 patients who were treated for <1 year (15). The long-responder group included 20 patients with SD and six with PR. Patients who received ivosidenib for ≥1 year had markedly prolonged PFS compared with those receiving shorter treatment (median 16.6 vs 1.8 months), with 68% of the long-responder group being progression-free at 12 months. The PFS rate in the long-term responder group was 69.2% at 12 months and 24.9% at 24 months. Most of the long-term responders were still alive after 2 years, with a 2-year OS rate of 92.1% in the long-term responders group versus 8.6% in the group who stopped ivosidenib within 1 year (15).

An anecdotal report of a durable response to ivosidenib, similar to the case described here, has been published. This involved two patients (an 80-year-old male and a 60-year-old female) with previously treated unresectable and metastatic *IDH1*-mutant iCCA. The patients achieved progression-free disease control lasting 13 and 20 months and a best response of SD and partial remission, respectively; ivosidenib was well tolerated (16). A real-world study of response to ivosidenib in 11 patients (54% female; median age 57 years) with previously treated locally advanced or metastatic *IDH*1-mutated CCA found median PFS of 4.4 months (95% CI 2.0–5.8) and OS of 15 months (95% CI 6.6–15.0), after a median follow-up of 13.7 months. The DCR rate was 63% and two patients achieved PR (17, 18). Again, ivosidenib was well tolerated, with two patients experiencing treatment-related adverse events (AEs), all of which were Grade 2. Two more recent real-world studies (n = 46 and 262) reported similar durable responses to ivosidenib, with median PFS of 3.7 months (95% CI 2.2–36.5), median OS of 11.5 months (95% CI9.5–36.5) and a DCR of 50% at median follow-up of 11.9 months in one study (19), and a median PFS of 4.7 months (95% CI 3.5–5.7), median OS of 15.5 months (95% CI 12.7–not estimable) and a DCR of 52% in the other study (20).

These data illustrate that, for some patients who respond to ivosidenib, the response can be durable. Like in this case, ivosidenib treatment allowed patients who experience minimal tolerability issues, to not only live longer, but to enjoy a good health-related quality of life (HRQoL).

## 3 Case 2

A 62-year-old Caucasian woman, with a history of epilepsy and bipolar disorder, presented with jaundice in February 2024 while travelling in Spain. A liver biopsy showed adenocarcinoma cells consistent with CCA. She underwent endoscopic retrograde cholangiopancreatography (ERCP) with brush cytology and stent placement in the common hepatic bile duct.

In March 2024, a CT of the thorax and abdomen showed multiple lesions in the liver suspicious for metastases, with the largest in segments VI and IVa (**Figure 2A**), and semicircular thickening in the walls of the gallbladder infundibulum (the suspected primary tumor). Stent patency was confirmed. Liquid biopsy was taken because there was insufficient tumor tissue in the biopsy and cytology specimens for mutation analysis. Molecular testing found an R132C mutation in *IDH1*, amplification of *HER2* and an L191fs*16 mutation in the gene for Fanconi anemia complementation group L (*FANCL*).

**Figure 2:**
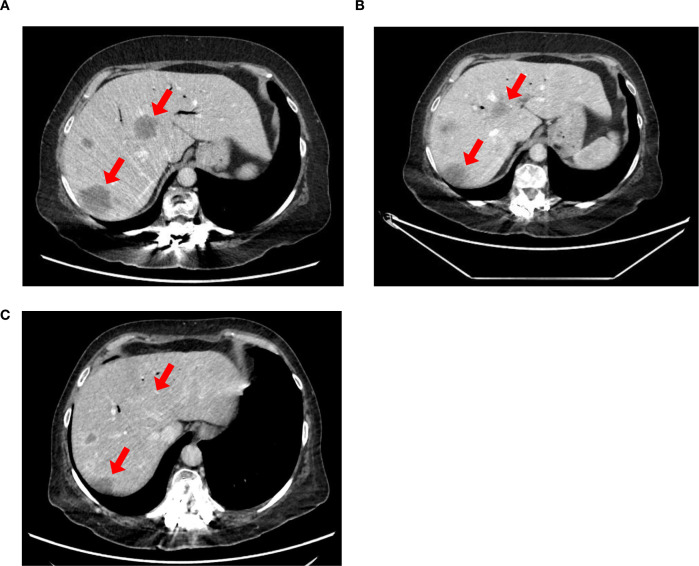
Abdominal computed tomography scans of case 2 at **(A)** first presentation in March 2024, **(B)** in July 2024 after five cycles of cisplatin + gemcitabine + durvalumab (prior to initiation of ivosidenib), and **(C)** in October 2024 after 3 months of treatment with ivosidenib, showing tumor shrinkage. Red arrows denote reference lesions.

The patient began first-line systemic chemotherapy with cisplatin + gemcitabine + durvalumab according to the TOPAZ-1 protocol (13). At 2 months (May 2024), CT imaging showed pre-existing multiple liver metastases with variable dynamics, i.e., partly size regressive and partly newly demarcated (e.g., segment II), a patent stent, and no change in the pre-existing prominent lymph node in the liver hilum. A follow-up CT scan in July 2024 showed SD (**Figure 2B**), but the patient experienced significant treatment-associated AEs, including immune-related toxicity with symptomatic hyperthyroidism (palpitations, tremor), nausea, vomiting, loss of appetite, and fatigue. After discussing the lack of treatment response, the AEs, and the fact that she had two actionable molecular targets, systemic chemotherapy was discontinued. The patient indicated a preference for oral therapy.

She began ivosidenib in July 2024; 3 months later, there was clear evidence on CT imaging of tumor shrinkage in the liver metastases (particularly the subcapsular lesion in segment VII, which shrank from 34 mm to 24 mm; a lesion in segment VIII also shrank, from 25 mm to 15 mm), no new hepatic lesions, and no extra-hepatic findings (**Figure 2C**). At last follow-up in January 2025, CT imaging indicated that the patient had SD compared with assessment in October 2024, with no signs of progression but no further significant tumor shrinkage (e.g. the lesion in segment VII was 20 mm and that in segment VIII was 13 mm). Therefore, she continued ivosidenib, which was associated with minimal AEs.

The patient fulfilled her dream of retiring in Spain, moving there permanently in March 2025. While she is no longer treated at Stadtspital Triemli, recent correspondence from her husband (December 2025) indicated that her current oncologists had switched her back to chemotherapy. This suggests that the patient’s disease had progressed, giving a total duration of response to ivosidenib of 1.5 years.

### 3.1 Comment

This case illustrates several issues relevant to the management of CCA. Firstly, obtaining a sample for biopsy from the biliary tree is technically challenging (often because of the tumor location) and there is often insufficient tissue in samples for both histological assessment and molecular testing (21). A liquid biopsy is a reasonable option for collecting cell-free circulating DNA for molecular testing rather than a second tissue biopsy, as liquid biopsy has a low failure rate and is minimally invasive (21).

This patient had an *IDH1* mutation in association with gallbladder cancer (GBC) as the primary tumor, which is extremely rare (22). The presence of an *IDH1* mutation was suggestive of iCCA, since these mutations are rare in eCCA (10) and almost never present in GBC (22, 23). On the other hand, *HER2* amplification occurs with similar frequency in both iCCA and eCCA (10), and is also present in 10–15% of GBCs (4). The common molecular alterations in GBC are *HER2*, epidermal growth factor receptor *(EGFR)*, phosphatidylinositol-4,5-bisphosphonate 3-kinase (*PIK3CA), KRAS*, *TP53*, *BRAF, ERBB3*, and phosphatase and tensin homolog (*PTEN)* (4, 23). In this patient, the presence of *HER2* amplification provided a second option for targeted therapy, a rare occurrence that adds to the complexity around treatment decisions. In this case, the patient preferred *IDH1*-based therapy with ivosidenib because it is administered orally. However, in the event of disease progression, HER2-directed therapy may be considered as third-line treatment.

There is increasing interest in defining gene clusters in biliary tract cancers and their prognostic significance. Wang and colleagues reported that *IDH1/2* mutations were mutually exclusive from *FGFR2* fusions or *KRAS, TP53*, or *SMAD4* mutations in patients with iCCA (24). They found that *KRAS*, *TP53*, and *SMAD4* gene mutations occurred together and were associated with large bile duct histology, high carbohydrate antigen 19-9 (CA 19-9) levels, and poor prognosis. On the other hand, pair-wise associations of *IDH1/2* with *BAP1* mutations or *FGFR2–BAP1* fusions were associated with a small bile duct histological phenotype, low CA 19–9 levels, and good prognosis. A better understanding of gene mutation clusters in biliary tract cancer may help predict patient prognosis, as well as potentially inform treatment options/combinations.

This patient had clear evidence of tumor shrinkage during ivosidenib treatment; most patients who stay on ivosidenib have SD. In the ClarIDHy study, only 3/124 patients (2%) in the ivosidenib arm had PR, while 63 (51%) had SD (overall DCR of 53%); time to response in each these three patients were 2.2, 5.5, and 8.3 months, indicating that response to ivosidenib may take >2 months to become apparent (11). In the current case, PR was visible at the first assessment, 3 months after starting ivosidenib. Duration of response in the three partial responders in the ClarIDHy study was 2.7, 2.8, and 11.0 months (11).

Finally, it is worthwhile remembering that, even if they are doing well, patients with biliary stents should be actively monitored to identify early signs of biliary obstruction or infection (2).

## 4 Case 3

An 84-year-old Asian woman with no relevant comorbidities presented in April 2024 with abdominal symptoms. She had a history of previous hepatitis B infection, but was negative for hepatitis C and alpha fetoprotein. CT imaging showed a large mass in liver segments VIII and V, measuring 10 × 8 cm and suggestive of cancer, but no lesions or metastases in the thorax, and no signs of osseus metastasis were visible on thoracic or abdominal imaging.

Liver biopsy showed malignant proliferation with areas of necrosis, and no areas in which the parenchyma was unaffected by carcinoma. Molecular testing found: a p.R132C mutation in exon 4 of *IDH1*; p.G44S mutation in exon 2 of the mediator complex subunit 12 (*MED12*) gene; and amplification of the MET proto-oncogene, receptor tyrosine kinase (*MET*; 8×), and cyclin dependent kinase 6 (*CDK6*; 7×) genes on chromosome 7. In May 2024, MRI confirmed a 10 cm malignant lesion in the right hepatic lobe, but no locoregional lymphadenopathy. The presumptive diagnosis was peripheral iCCA.

Because of the patient’s advanced age and relative frailty (ECOG PS of 3), the decision was made to initiate first-line targeted therapy, and she began ivosidenib treatment as monotherapy (off-label treatment). Four months later (in September 2024), thoraco-abdominal CT showed a poorly defined, hypodense mass in the right lobe of similar or slightly smaller size compared with pre-treatment imaging (7 × 9.8 cm vs 8.1 × 10 cm at pre-treatment). There was no visible lymph node involvement and no evidence of suspicious lesions, including in bone.

Her clinical condition improved during ivosidenib treatment; prior to treatment she had been unable to walk outside, but after 1 month, she was able to work in the garden for 1 hour, and after 2 months, she went for a swim. During ivosidenib treatment, the patient consistently maintained an ECOG PS of 1, with no tumor-related pain, an increased appetite, and weight gain of 4–5 kg. CT scans in March 2025 and September 2025 found tumor shrinkage (to 5.7 × 8.8 cm and 4.3 × 6.4 cm, respectively).

However, in June 2024 she had presented to the emergency room with severe fatigue, vertigo, constipation, and nausea. Further diagnostic testing showed severe hypercalcemia with albumin-corrected levels of 3.87 mmol/L and secondary acute renal impairment with elevated levels of parathyroid hormone-related protein (PTHrP) and decreased levels of parathyroid hormone, indicating paraneoplastic hypercalcemia. An electrocardiogram (ECG) showed no cardiac arrhythmia. The patient received denosumab, calcitonin, and hydration, with subsequently decreasing calcium levels (to 2.32 mmol/L). Denosumab was administered once a month (without a change in dose or administration frequency), after which calcium levels normalized and clinical symptoms resolved. Signs of severe hypercalcemia such as constipation, vertigo, and nausea were almost immediately resolved with decreasing calcium levels during hospitalization. Her fatigue also improved significantly as calcium levels normalized, although there were other contributing factors to fatigue such as the carcinoma and its respective treatment, thus fatigue was not expected to resolve entirely. After 6 months of denosumab treatment, decreasing tumor activity, and normalized calcium levels, the treatment was able to be stopped (in January 2025). At the patient’s most recent assessment (January 2026), her calcium level was 2.47 mmol/L.

Treatment with ivosidenib is ongoing. The patient has reported very good quality of life and no signs of toxicity. Next follow-up is planned for March 2026.

### 4.1 Comment

In Western countries, CCA is a disease with a median age at onset of ~65 years, rarely occurring in individuals aged <50 years (25). Therefore, clinicians must consider patient comorbidities and ECOG PS when selecting treatment.

Ivosidenib is not approved for the first-line treatment of CCA, but has been approved in the United States and Europe for the first-line treatment of acute myeloid leukemia in patients who are not suitable candidates for chemotherapy based on age and comorbidities (26, 27). This prompted initiation of first-line ivosidenib as off-label treatment in this case, for whom there were no labelled treatment options due to her age and frailty (aged >80 years and ECOG PS of 3). Indeed, there is no current guidance from ESMO on risk-benefit considerations when prioritizing targeted therapy over standard chemo-immunotherapy in frail patients. While encouraging results were seen in case 3, widespread clinical use of ivosidenib in this manner will be contingent on results from clinical trials and specific regulatory approval. Targeted therapy has been useful as a first-line treatment in other tumor types. For example, in *EGFR*-mutated non-small cell lung cancer, osimertinib may be given alone or in combination with chemotherapy (28), and has also shown promise in this setting as monotherapy in patients with poor PS (29).

The current ESMO guidelines recommend conducting molecular testing at CCA diagnosis (2); this allows physicians to have the results ready for second-line treatment decisions without delaying the initiation of systemic chemotherapy. Conducting molecular testing once the disease has progressed introduces an unnecessary and potentially harmful treatment delay. In order for targeted therapies, like ivosidenib, to be used as first-line treatment, physicians must be confident that the delay in molecular testing results will not unduly compromise clinical outcomes for the patient, or (as in case 3) that the potential benefits outweigh any potential risks.

The ESMO guidelines highlight the importance of best supportive care for patients with advanced and/or metastatic disease (2). This is arguably even more important for patients with poor PS. Patients with ECOG PS 3 (as case 3 was prior to treatment) are usually excluded from clinical trials (such as TOPAZ-1), so the safety and tolerability of targeted treatments in this vulnerable population are unknown (13). In the TOPAZ-1 study, in which the study population was limited to patients with an ECOG PS of 0 or 1, >75% of patients who received first-line chemotherapy with cisplatin + gemcitabine with durvalumab or placebo developed a grade 3 or 4 AE, and 4% in either treatment group died due to an AE (13). In contrast, only 6% of patients in the ivosidenib arm of the ClarIDHy study developed a grade ≥3 treatment-related AE, and none of the deaths in the study were caused by treatment-related AEs (11).

While most AEs associated with ivosidenib are easily managed, physicians need to be aware of the potential for QT prolongation and ensure that patients undergo ECG prior to initiating ivosidenib, at least weekly for the first 3 weeks of treatment, and monthly thereafter (26, 27). During ivosidenib treatment, the patient in case 3 developed malignant hypercalcemia, which is a relatively common complication of cancer caused by the excessive secretion of PTHrP by tumor cells (30). Approximately 30% of patients with CCA will develop malignant hypercalcemia over the course of their disease, and the prevalence is higher in patients with an *IDH1* mutation than in those with wild-type *IDH1* (31). Tumor characteristics associated with an increased prevalence of hypercalcemia are high tumor burden (iCCA) and poor tumor differentiation (eCCA) (31). Late development of hypercalcemia (>70 days after diagnosis) and older age are risk factors for a poor outcome (32). It is important to treat malignant hypercalcemia promptly because it can have a negative impact on prognosis, but many symptoms (abdominal pain, nausea, and appetite loss) are non-specific and difficult to distinguish from cancer-related symptoms (33). The usual approach to treatment is intravenous hydration, calcitonin, and bisphosphonates (30). Denosumab is an appropriate alternative to bisphosphonates in patients with renal impairment, since it is not renally excreted (30).

CCA has a significant effect on the HRQoL of patients and their caregivers, with a greater impact observed in patients aged >45 years (34). In the ClarIDHy study, the physical function domain of HRQoL declined in both the ivosidenib and placebo arms, but the rate of decline was significantly greater with placebo than with ivosidenib, indicating that ivosidenib may preserve physical function and HRQoL in patients with advanced CCA (11). In case 3, the preservation of physical function with ivosidenib was exemplified by the improvement in ECOG PS and the ability of the patient to engage in activities she enjoyed.

## 5 Discussion

While late-phase clinical studies, such as ClarIDHy (11), provide large quantities of robust data, the very controlled methodology and strict inclusion/exclusion criteria may limit the generalizability of their findings to many real-world patient populations. Data regarding the use of ivosidenib as second- and later- line therapy for advanced *IDH1*-mutant CCA in real-world settings are beginning to be published (17–20). The cases reported in the current manuscript add to knowledge regarding the real-world use of ivosidenib, describing its effects in the longer-term (case 1), the challenges of treatment choice in the rare incidence of the presence of more than one actionable target (case 2), and off-label use (i.e. as first-line treatment) in a frail older patient (case 3); thus, providing useful insights to guide real-word clinical use of the drug.

Currently, ivosidenib is not approved for the first-line treatment of CCA, but it is likely that the use of ivosidenib and other targeted therapies will evolve and expand over time. An analysis of recent trends in research into targeted therapy for CCA found an abundance of literature on growth factor receptors (35), suggesting that targeting these regulators of cellular proliferation, differentiation and migration is an ongoing focus in the search for new treatments for CCA. Indeed, current studies are investigating the earlier use of ivosidenib and other targeted therapies in the treatment paradigm for CCA (**Table 1**). The SAFIR-ABC study (NCT05615818) is investigating whether patients who achieve disease control after four cycles of standard first-line chemotherapy, and have at least one targetable mutation, can be switched to maintenance targeted therapy or whether they should complete the usual eight cycles of chemotherapy. A phase 1/2 study (NCT06501625) is investigating whether adding ivosidenib to the standard first-line gemcitabine + cisplatin + durvalumab regimen improves clinical outcomes in participants with an *IDH1* mutation with locally advanced or metastatic CCA; the primary endpoint is safety in the lead-in phase and ORR in the expansion phase. This study will include patients who have received one cycle of chemotherapy, meaning that patients receive treatment while waiting for the results of molecular testing (36).

**Table 1:** Ongoing or recently completed studies investigating ivosidenib in cholangiocarcinoma.

Clinicaltrials.gov identifier	Phase	Patient population	N	Treatment(s)	Primary endpoint	Trial status
NCT04088188	1	Advanced (nonresectable or metastatic) CCA with no evidence of progression after the first 3 cycles of cisplatin + gemcitabine and either *IDH1* mutation or *FGFR2* gene alteration	8^a^	Ivosidenib or pemigatinib in combination with cisplatin + gemcitabine	Significant (dose-limiting) toxicities	Terminated (December 2023, low accrual), initial results posted on ClinicalTrials.gov
NCT05921760	1/2	Advanced (nonresectable or metastatic) CCA and *IDH1* mutation	7^a^	Ivosidenib + nivolumab + ipilimumab	Safety phase: Significant (dose-limiting) toxicities and recommended combination dose Expansion phase: ORR	Completed (November 2024), results awaited
NCT05876754	3b	Previously treated advanced (nonresectable or metastatic) CCA and *IDH1* mutation	220^b^	Ivosidenib	Safety	Recruiting (estimated completion, June 2025)
NCT06081829	2	Previously treated advanced (nonresectable or metastatic) CCA and *IDH1* mutation	12^a^	Ivosidenib	6-month PFS (using RECIST v1.1)	Active, not recruiting (estimate completion, May 2027)
NCT06501625	1b/2	Previously untreated advanced (nonresectable or metastatic) CCA and *IDH1* mutation	52^b^	Ivosidenib + durvalumab + gemcitabine + cisplatin	Safety phase: Significant (dose-limiting) toxicities, AEs, including AEs of special interest and serious AEs Expansion phase: ORR	Ongoing (estimated completion, September 2027)
NCT06607302	N/A^c^	Previously treated locally advanced or metastatic CCA and *IDH1 R132* mutation	100^b^	Ivosidenib	PFS	Recruiting (estimated completion, December 2027)
NCT07260175	2	Previously treated (adjuvant systemic SOC chemotherapy) curatively resected *IDH1*-mutated iCCA, without metastatic spread	40^b^	Ivosidenib maintenance	1-year recurrence-free survival rate	Recruiting (estimated completion, December 2031)

^a^ Actual enrolment; ^b^Estimated enrolment; ^c^Observational real-world study.

AE, adverse event; CCA, cholangiocarcinoma; *FGFR*, fibroblast growth factor receptor; iCCA, intrahepatic cholangiocarcinoma; *IDH1*, isocitrate dehydrogenase 1; ORR, objective response rate; PFS, progression-free survival; RANO, Response Assessment in Neuro-Oncology; RECIST, Response Evaluation Criteria in Solid Tumors; SOC, standard of care.

The increased use of targeted therapy means that physicians need to be aware of the need for early molecular testing, and how to interpret the results. CCA is a rare disease, and most actionable driver mutations occur in only a small number of patients (10), so most physicians outside of tertiary centers will treat CCA in patients with a targetable mutation irregularly. Therefore, there is an opportunity to improve the management of CCA by creating networks of expert centers to disseminate accurate and clinically practical information more widely through established and novel educational initiatives (37).

In conclusion, since molecular targeted therapies are the recommended second-line treatment option for patients with CCA with an actionable driver mutation who progress after first-line systemic chemotherapy, early molecular testing is essential to guide second-line treatment decisions. This ensures that there is no delay between the end of first-line chemotherapy and the initiation of second-line treatment. As illustrated by these three cases, some patients with CCA and *IDH1* mutations derive prolonged benefits from ivosidenib treatment, both in terms of disease control and HRQoL, and can maintain long-term treatment because the tolerability profile is generally moderate and manageable. In the future, it is likely that the role of ivosidenib and other targeted therapies in CCA management will evolve to be included first-line therapy regimens and in novel combinations.

## Data Availability

The original contributions presented in the study are included in the article/**Supplementary Material**. Further inquiries can be directed to the corresponding author.
