# Supplementary material for: Case Report: Response to ivosidenib in patients with cholangiocarcinoma: a clinical perspective with illustrative cases
Source: Front Oncol. 2026 Jun 2;16:1617664. doi: 10.3389/fonc.2026.1617664 (PMC13270435; doi:10.3389/fonc.2026.1617664)
Supplement: Supplementary file 1 [file DataSheet1.pdf]

Supplementary Material

1 **Supplementary Table S1.** Molecular targets determining second-line treatment options in European Society for Medical Oncology guidelines [1].

**Molecular marker**	**Frequency**	**Detection method**	**ESCAT score**	**ESMO-recommended therapy**	**Level of evidence**	**MCBS v1.1 score**
IDH1 mutation	1–18%	NGS	I-A	Ivosidenib	IA	2
FGFR2 fusions or rearrangements	<5%	RNA sequencing	III-A	Futibatinib	IIIA	3
				Pemigatinib	IIIA	2
BRAF^{V600E} mutation	<5%	NGS	I-B	Dabrafenib-trametinib	IIIA	3
HER2 amplifications and overexpression	5–10%	NGS, FISH, IHC	I-C	Trastuzumab deruxtecan	IIIA	3
				Zanidatamab	IIIA	3
NTRK fusions	<1%	RNA sequencing	I-C	Entrectinib	IIIA	3
				Larotrectinib	IIIA	3
				Repotrectinib	IIIA	–
RET fusions	<1%	RNA sequencing	I-C	Selpercatinib	IIIA	3
BRCA1/2 mutations	3–5%	NGS	III-A	PARP inhibitors (e.g., olaparib)	VB	–
PALB2 mutations	1%	NGS	III-A	PARP inhibitors (e.g., rucaparib)	VB	–
MSI-H/dMMR	<1%	IHC	I-C	Pembrolizumab	IIIA	3

BRAF, proto-oncogene B-Raf; *BRCA*, breast cancer gene; dMMR, mismatch repair deficiency; ESCAT, ESMO Scale for Clinical Actionability of molecular Targets; ESMO, European Society for Medical Oncology; *FGFR*, fibroblast growth factor receptor; FISH, fluorescence *in situ* hybridization; *HER2*, human epidermal growth factor receptor 2; *IDH1*, isocitrate dehydrogenase 1; IHC, immunohistochemistry; MCBS, Magnitude of Clinical Benefit Scale; MSI-H, microsatellite instability – high; NGS, next-generation sequencing; *NTRK*, neurotrophic tyrosine kinase; *PALB2*, partner and localizer of BRCA2; PARP, poly ADP-ribose polymerase; *RET*, rearranged during transfection.

ESCAT categories: I-A, alteration–drug match is associated with improved outcomes, with evidence from randomized clinical trials showing the alteration–drug match in a specific tumor type results in a clinically meaningful improvement of a survival endpoint; I-B, alteration–drug match is associated with improved outcomes, with evidence from prospective, nonrandomized clinical trials showing that the alteration–drug match in a specific tumor type results in clinically meaningful benefit, as defined by ESMO-MCBS v1.1; I-C, alteration–drug match is associated with improved outcomes, with evidence from clinical trials across tumor types or basket clinical trials showing clinical benefit associated with the alteration–drug match, with similar benefit observed across tumor types; II-B, alteration–drug match is associated with antitumor activity, with evidence from prospective clinical trials showing the alteration–drug match in a specific tumor type results in increased responsiveness when treated with a matched drug; however, no data are currently available on survival endpoints; III-A, alteration–drug match is suspected to improve outcomes based on patients with the specific alteration but in a different tumor type, with limited/absence of clinical evidence available for the patient-specific cancer type or broadly across cancer types.

2 References

[1] Vogel A, Ducreux M, on behalf of the ESMO Guidelines Committee. ESMO Clinical Practice Guideline interim update on the management of biliary tract cancer. ESMO Open 2025;10:104003.
